# cGMP-dependent protein kinase from Toxoplasma gondii: functional expression in E. coli and molecular characterization

**DOI:** 10.1186/1471-2210-11-S1-P45

**Published:** 2011-08-01

**Authors:** Caitlin J McFarland, Christian K Nickl, Brent W Osborne, Indra Neil Sarkar, Wolfgang R Dostmann

**Affiliations:** 1 Department of Pharmacology, College of Medicine, University of Vermont, Burlington, VT 05405, USA; 2 Center for Clinical & Translational Science, Department of Microbiology & Molecular Genetics and Department of Computer Science, University of Vermont, Burlington, VT 05405, USA

## Background

The apicomplexan parasite *Toxoplasma gondii* is an obligate intracellular human pathogen causing toxoplasmosis predominantly in immune-compromised hosts such as cancer and transplant patients as well as patients with AIDS [1]. A specific cGMP-dependent protein kinase (*Tg*PKG) which appears to be crucial for host invasion has been identified in *T. gondii* and related coccidial protozoa [2]. However, detailed structural and biochemical analyses have been hampered due to the inability to functionally express these kinases in high yields in systems other than their parasite host organisms.

## Results

Here we describe the expression, purification and initial characterization of the 911 amino acid (103 kDa) His-tagged type II isoform of *Tg*PKG using a bacterial source. A phylogenetic analysis further reveals that *Tg*PKG2 belongs to an evolutionarily distinct sub-group of the AGC-kinase family. The overall domain composition of *Tg*PKG2 substantially deviates from its mammalian cousins at two regions. First, the 196 amino acid N-terminal/auto-inhibitory domain bears no resemblance with any other PKG subfamily, and secondly the kinase consists of three cGMP binding sites with the third binding sites separated from the others by 135 amino acids. Consequently, *Tg*PKG2 illustrated a remarkable level of cooperativity (n_H_ = 2.9) accompanied by a 200-400 fold cGMP-mediated activation utilizing the common PKG substrate TQAKRKKSLAMA (K*_m_* = 9 µM). The associated activation constant was 1.7 µM which is in full agreement with the isoforms obtained from *T. gondii* parasite extract [3]. Interestingly, *Tg*PKG2 was completely insensitive to cAMP (K*_a_* » 100 µM). Recently, the trisubstituted pyrrole 4-[2-(4-fluorophenyl)-5-(1-methylpiperidine-4-yl)-1H pyrrol-3-yl] pyridine (Compound 1) was shown to exhibit anticoccidial kinase activity [3,4]. Compound 1 blocked kinase activity of *Tg*PKG2 with high potency (IC_50_ = 59 nM) and high selectivity; the mammalian type Iα PKG showed an approximately 1000-fold reduced IC_50_ of 45 µM.

## Conclusion

This work demonstrates the first catalytically active expression of any cGMP-dependent protein kinase from *E. coli* and may provide a new platform for the functional and structural analysis, as well as evolutionary history, of PKG isoforms from apicomplexan parasites.
